# Hot-melt Adhesive Bonding of Polyurethane/Fluorinated Polyurethane/Alkylsilane-Functionalized Graphene Nanofibrous Fabrics with Enhanced Waterproofness, Breathability, and Mechanical Properties

**DOI:** 10.3390/polym12040836

**Published:** 2020-04-06

**Authors:** Chunhui Liu, Xi Liao, Weili Shao, Fan Liu, Bin Ding, Gaihuan Ren, Yanyan Chu, Jianxin He

**Affiliations:** 1 College of Textile, Zhongyuan University of Technology, Zhengzhou 450007, China; lch1820921715@163.com (C.L.); xiliao1208@163.com (X.L.); WeiliShao@163.com (W.S.); liufan365@163.com (F.L.); 2 Key Laboratory of Textile Science & Technology, Ministry of Education, College of Textiles, Donghua University, Shanghai 201620, China; binding@dhu.edu.cn

**Keywords:** waterproof-breathable, electrospinning, hydrophobic, sheet structure, fusion structure, hot-press treatment

## Abstract

Waterproof-breathable (WB) materials with outstanding waterproofness, breathability, and mechanical performance are critical in diverse consumer applications. Electrospun nanofibrous membranes with thin fiber diameters, small pore sizes, and high porosity have attracted significant attention in the WB fabric field. Hot-press treatment technology can induce the formation of inter-fiber fusion structures and hence improve the waterproofness and mechanical performance. By combining electrospinning and hot-press treatment technology, polyurethane/fluorinated polyurethane/thermoplastic polyurethane/alkylsilane-functionalized graphene (PU/FPU/TPU/FG) nanofiber WB fabric was fabricated. Subsequently, the morphologies, porous structure, hydrostatic pressure, water vapor transmission rate (WVTR), and stress–strain behavior of the nanofiber WB fabric were systematically investigated. The introduction of the hydrophobic FG sheet structure and the formation of the inter-fiber fusion structure greatly improved not only the waterproofness but also the mechanical performance of the nanofiber WB fabric. The optimized PU/FPU/TPU-50/FG-1.5 WB fabric exhibited an excellent comprehensive performance: a high hydrostatic pressure of 80.4 kPa, a modest WVTR of 7.6 kg m^−2^ d^−1^, and a robust tensile stress of 127.59 MPa, which could be used to achieve various applications. This work not only highlights the preparation of materials, but also provides a high-performance nanofiber WB fabric with huge potential application prospects in various fields.

## 1. Introduction

Waterproof-breathable (WB) fabrics are materials that have waterproof, breathable, windproof, and warmth-generation properties [1,2,3]. They can be classified as high-density, coated, and laminated fabrics [4,5,6,7,8], according to the processing technology. Compared with high-density and coated fabrics, laminated fabrics can easily achieve improved waterproofness and breathability, synchronously. However, the waterproofness, and durability of laminated fabrics are deteriorated by the stretching of the fabric during usage. Therefore, to achieve the durability of WB laminated composite fabrics, it was imperative to endow the fabric with waterproofness, breathability, and mechanical performances.

Nanofiber membranes prepared by electrospinning technology possess fine fiber diameters, high porosity, small pore sizes, and interconnected porous structures, which afford them the ability to prevent the penetration of liquid droplets yet allow gas or vapor to pass through [9,10,11,12,13,14]. Based on these characteristics, electrospinning technology is widely employed to prepare nanofiber WB membranes. A variety of polymers, such as polyurethane (PU) [15], nylon [16], polyacrylonitrile (PAN) [17], polyvinylidene fluoride (PVDF) [18], and polytetrafluoroethylene (PTFE) [19], have been utilized in the preparation of nanofiber WB membranes. Among these nanofibers, PU nanofibers are the most widely used due to their attractive characteristics, such as good elasticity, high durability, easy-care, and good comfort properties. Park et al. [20] first prepared PU nanofiber WB membranes through electrospinning, which displayed poor water resistance (hydrostatic pressure, 3.7 kPa) and good breathability (water vapor transmission rate (WVTR), 9 kg m^−2^ d^−1^).

Outstanding hydrophobicity is needed to obtain high hydrostatic pressure. Adding chemicals with hydrophobic long perfluorinated carbon chains can help improve the hydrophobicity of the fiber membrane and, thereby, improve its water resistance. By introducing hydrophobic fluorinated polyurethane (FPU) into the PU, Ge et al. [21] obtained PU/FPU nanofiber WB membranes, which exhibit enhanced water resistance compared with pure PU nanofiber WB membranes (hydrostatic pressure, 39.3 kPa vs 3.7 kPa) and remained good breathability (WVRT, 10.9 kg m^−2^ d^−1^). However, due to the general waterproofness and poor mechanical performance of the electrospun PU/FPU nanofiber WB membranes, they could not satisfy the requirements for practical application [22,23,24]. Presently, researchers have attempted to improve the waterproofness and mechanical performance of nanofiber membranes by blending low-melting-point polymers combined with heat treatment. Sheng et al. [25] subjected the PAN/FPU nanofiber membrane to heat treatment, which greatly improved its waterproofness (hydrostatic pressure, 114.6 kPa) and mechanical performance (breaking strength, 9.4 MPa). This was due to the fusion of low-melting-point PAN components under the treatment temperature (90 °C). However, the study did not investigate the combination of the nanofiber membrane with fabric. The effective combination of the nanofiber membrane with base fabric into a nanofiber WB fabric is crucial for practical applications. More importantly, the introduction of hydrophobic material and the proper inter-fiber fusion structure can endow the nanofiber WB fabric with the superior waterproofness, breathability, and mechanical performance. 

In this work, a nanofiber WB fabric with the desired waterproofness, breathability, and mechanical performance was prepared. Here, hydrophobic hexadecyltrimethoxysilane-functionalized graphene oxide (FG, hydrophobic agent) and low-melting thermoplastic polyurethane (TPU, fiber binders) were introduced into PU/FPU electrospinning solutions. The introduction of the hydrophobic FG sheet structure and the formation of the inter-fiber fusion structure by TPU improved not only the waterproofness but also the mechanical performance of the nanofiber WB fabric, which would be an appropriate candidate in various applications, especially for fabricating of protective garments and self-cleaning materials [26,27,28].

## 2. Materials and Methods

### 2.1. Materials

PU was obtained from BASF. FPU was purchased from Jiangsu Baoze polymer material Co., Ltd (Taicang, China). TPU was obtained from Shenzhen Junsu Co., Ltd (Shenzhen, China). The base fabric is polyester fabric with the warp and weft yarn density of 68 D × 68 D, and the fabric gram weight of 200 g m^−2^, and purchased from Wujiang Qiading Textile Co., Ltd (Wujiang, China). Graphene oxide (GO) was purchased from Suzhou Tanfeng graphene technology Co., Ltd (Suzhou, China). Hexadecyltrimethoxysilane (HDTMS) was obtained from Aladdin Chemical Reagents Co., Ltd (Shanghai, China). Triethylamine (TEA), *N,N*-dimethylformamide (DMF), N,N-dimethylacetamide (DMAc), acetone, and ethanol were provided by the China Pharmaceutical Group Chemical Reagents Co., Ltd (Shanghai, China). All the chemicals were of analytical grade and were used without further purification.

### 2.2. Preparation of FG

FG was prepared as previously reported [29]. First, GO (200 mg) was dispersed in DMF (100 mL) under ultrasonic conditions. Thereafter, TEA (0.3 mL) and HDTMS (2.7 g) were added into the GO dispersion. After stirring for 30 min, the mixture was transferred into a three-necked bottle and refluxed at 110 °C under the protection of nitrogen for 24 h. The dispersion was washed four times with ethanol by repeated centrifugation. The product was dried in a vacuum oven at 60 °C for 24 h to obtain hydrophobic FG.

### 2.3. Preparation of PU/FPU/TPU-50/FG Nanofiber WB Fabric

The TPU solution (22 wt %) was prepared by dissolving TPU in a mixture of DMAc/acetone (4:6 *w*/*w*) with stirring for 3 h. FG was dispersed in the mixture of DMAc/acetone (4:6 *w*/*w*) by ultrasonication for 4 h. The PU/FPU/TPU/FG solution with a concentration of 14.5 wt % was obtained by dissolving PU, FPU, TPU and FG in the mixture of DMAc/acetone (4:6 *w*/*w*). The weight ratio of PU:FPU was fixed at 9:1, PU/FPU/TPU hybrids with 50 wt % TPU, with respect to the ratio of PU, were designated as PU/FPU/TPU-50, and the PU/FPU/TPU-50/FG hybrids with 0.5, 1.0, and 1.5 wt % FG, with respect to the ratio of PU/FPU/TPU-50, were designated as PU/FPU/TPU-50/FG-0.5, PU/FPU/TPU-50/FG-1.0, PU/FPU/TPU-50/FG-1.5, and PU/FPU/TPU-50/FG-2.0, respectively. The thin TPU hot-melt adhesion layer was sprayed onto the fabric, and the PU/FPU/TPU-50/FG nanofibers were collected on the fabric covered with the hot-melt adhesion layer. The voltage applied to the needle tip, the receiving distance, the flow rate and electrospinning duration were 25 kV, 18 cm, 0.1 mm/min, and 2 h, respectively. The ambient temperature and relative humidity were 28 ± 2 °C and 30% ± 2%, respectively. The thickness of the nanofiber membrane fabric was 120−130 μm. The hot-press treatment was used to prepare the nanofiber WB fabrics. The winding speed of the hot-press roller was fixed at 3.02 r/min, and the treatment temperature was conducted at 120 °C.

### 2.4. Preparation of the PU/FPU/TPU Nanofiber WB Fabric

The TPU solution (22 wt %) was prepared by dissolving TPU in a mixture of DMAc/acetone (4:6 *w*/*w*) with stirring for 3 h. The PU/FPU/TPU solution with a concentration of 14.5 wt % was obtained by dissolving PU, FPU, and TPU in the mixture of DMAc/acetone (4:6 *w*/*w*). The weight ratio of PU:FPU was fixed at 9:1, and PU/FPU/TPU hybrids with 0, 25, 50, and 75 wt % TPU, with respect to the ratio of PU, were designated as PU/FPU, PU/FPU/TPU-25, PU/FPU/TPU-50, and PU/FPU/TPU-75, respectively. The spinning process was the same as that aforementioned. The winding speed of the hot-press roller was fixed at 3.02 r/min, and the treatment temperature was conducted at 80, 100, 120, and 140 °C.

### 2.5. Materials Characterization

The structure and morphology of the nanofiber membranes were observed by SEM (Phenom, PA, USA) and TEM (JEM-2100, Japan Electronics, Tokyo, Japan). The pore size of the nanofiber membrane was tested using a capillary aperture analyzer (CFP-1100AI, PMI company, Oregon, USA). The thickness of the nanofiber membrane fabric was measured using a digital display thickness gauge (CHY-C2, Languang Technology Corporation, Yiwu, China), and the average value was obtained by 20 measurements. A model OCA20 contact angle meter (Dataphysics, Filderstadt, Germany) was employed to measure the contact angles of the nanofiber membrane with 5 μL of water.

The porosity of all the nanofiber membranes was calculated using the following equation:

(1) $$\mathrm{Porosity}=(1\;-\;\frac{m}{t\;\times \;S\;\times \;\rho })\;\times \;100\%$$

where m, t, and S are the mass, thickness, and area of per unit measured membrane, respectively, and ρ is the density of the polymer raw material (the density value of the composite membrane is the weighted sum of the density mass fractions of its raw materials).

### 2.6. Materials Performance Testing

The waterproof and breathable performance of the nanofiber WB fabric was evaluated for hydrostatic pressure, water vapor transmittance, and air permeability. The hydrostatic pressure of the nanofiber WB fabric was measured using a YG812F water permeability tester. Water vapor transmittance measurement was carried out using the water vapor transmittance tester (model W3/031, Jinan Languang electromechanical technology Co., Ltd., Jinan, China). The sample was covered over a permeable cup containing deionized water and, subsequently, placed in a balanced permeable box at 38 °C and 90% relative humidity.

The WVTR was calculated according to the following equation:

(2) $$\mathrm{WVTR}=\frac{G}{tA}\;\times \;24,$$

where WVTR is expressed in kg m ^−2^ d^−1^, G is the weight change of the test cup, t is the time change of the test, and A is the measuring area of the sample. The air permeability of the covered sample over a permeable cup containing deionized water was tested using a YG461E-III air permeability tester (Wenzhou jigao testing instrument Co. LTD, Wenzhou, China).

The tensile strength of the nanofiber WB fabric was examined using a fiber strength tester (LILY-06ED/PC, Jinan languang electromechanical technology Co. LTD, Jinan, China). The sample with the same weight was cut into slivers with lengths of 100 mm and widths of 3 mm. The clamping length was 50 mm, and the tensile speed was 50 mm/min. Each sample was tested 20 times to calculate the average strength of the membrane fabrics.

## 3. Results and Discussion

### 3.1. Preparation of the Nanofiber WB Fabrics

**Figure 1:**
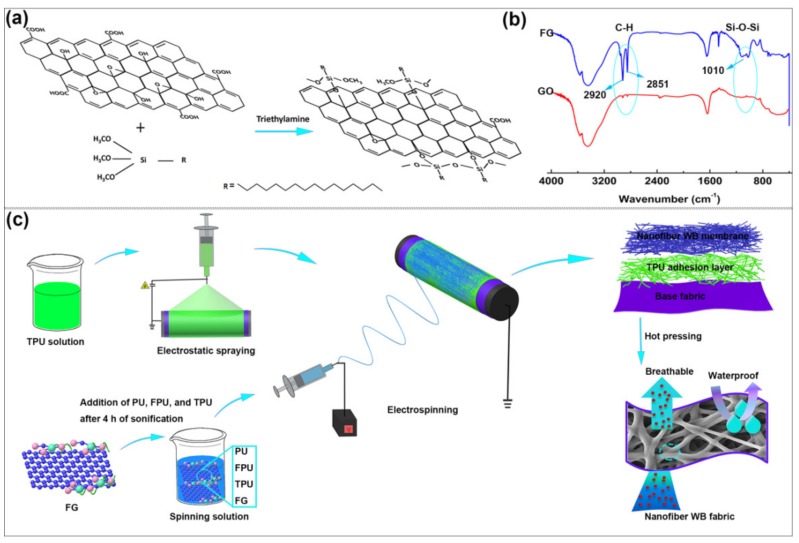
(**a**) The synthesis of functionalized graphene (FG); (**b**) FTIR spectra of graphene oxide (GO) and FG; (**c**) illustration of the fabrication process of a polyurethane (PU)/polyurethane/fluorinated (FPU)/polyurethane/thermoplastic (TPU)/FG nanofiber WB fabric.

We prepared firstly FG by functionalization of GO with HDTMS. As a silane coupling agent, HDTMS not only react with hydroxyl groups on the surface of GO, but also reduce the surface energy of FG owning to the introduced hydrophobic long chain alkyl (Figure 1a). The FTIR spectra of GO and FG are shown in Figure 1b. Compared to the spectrum of GO, new double peaks at 2920 and 2851 cm^−1^, corresponding to symmetrical and asymmetrical C–H stretching vibrations, appear in the spectrum of FG. In addition, the peak at 1010 cm^−1^ was assigned to the vibration of Si–O–Si in the spectrum of FG [30,31]. These spectral characteristics indicate that HDTMS molecules are successfully attached onto the GO, and therefore indicate the successful synthesis of FG. Then, as shown in Figure 1c, the PU/FPU/TPU/FG nanofiber membrane was collected on the fabric covered with a hot-melt adhesion layer (TPU layer), the TPU layer was used as adhesive agent to combine the nanofiber membrane with the base fabric. Finally, the nanofiber WB fabrics with hydrophobic FG sheet structures and inter-fiber fusion structures were obtained by applying the hot-press treatment to the nanofiber WB membrane-TPU adhesion layer-base fabric. 

### 3.2. Microstructure and SURFACE Wettability of PU/FPU/TPU-50/FG Nanofiber WB Fabrics

**Figure 2:**
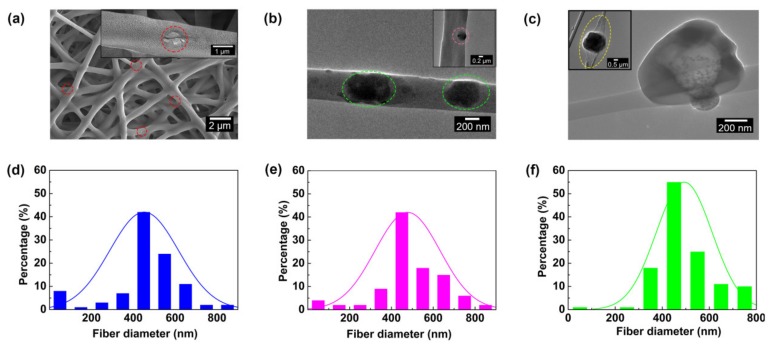
(**a**) SEM image of PU/FPU/TPU-50/FG WB fabrics with 1.0 wt % FG; (**b**) and (**c**) TEM images of PU/FPU/TPU-50/FG WB fabrics with 1.0 wt % FG; Diameter distribution diagram of PU/FPU/TPU-50/FG nanofiber WB fabrics at different FG concentrations: (**d**) 1.0, (**e**) 1.5, and (**f**) 2.0 wt % FG.

Figure 2a−c shows the morphologies of WB fabrics. As can be observed from the SEM image and its enlarged illustration (Figure 2a, red coil), the FG sheet was doped into the nanofiber, and the majority of the FG sheet was in the single nanofiber. To further demonstrate the distribution of the FG sheet inside the nanofiber membrane, it was characterized by TEM (Figure 2b,c). The FG sheet adhered to the surface of the single nanofiber and was covered with a layer of blended polymer (indicated with the green dotted line in Figure 2b). However, the FG sheet was not completely blended in a single nanofiber. Fragments of the FG sheet were exposed to air and were covered by a layer of polymer (indicated with the pink dotted line in Figure 2b). Moreover, in some cases, most of the FG sheets were distributed outside the single fiber (Figure 2c), indicating that the FG sheet would overlap at the breakpoint when the nanofiber breaks. The above-mentioned distribution and structure of FG can provide effective support for changes of waterproof, breathable and mechanical performances of the nanofiber WB fabrics [32,33].

Considering the diameter distribution of nanofiber could affect the pore structure of PU/FPU/TPU-50/FG WB fabrics, the statistical analysis results of the SEM image are investigated (Figure S1, Figure 2d–f). The increment of the FG content from 0.5 to 2.0 wt % resulted in only a slight increase in the nanofiber average diameter from 440.0 to 493.2 nm, but in a significant decrease in particle size distribution. The decrease in particle size distribution indicates that nanofiber diameter distribution tends to be uniform. The more uniform the nanofiber diameter was, the easier to fill the pores evenly, and the uniform pores could effectively prevent water droplets from penetrating into the fabric [34].

**Figure 3:**
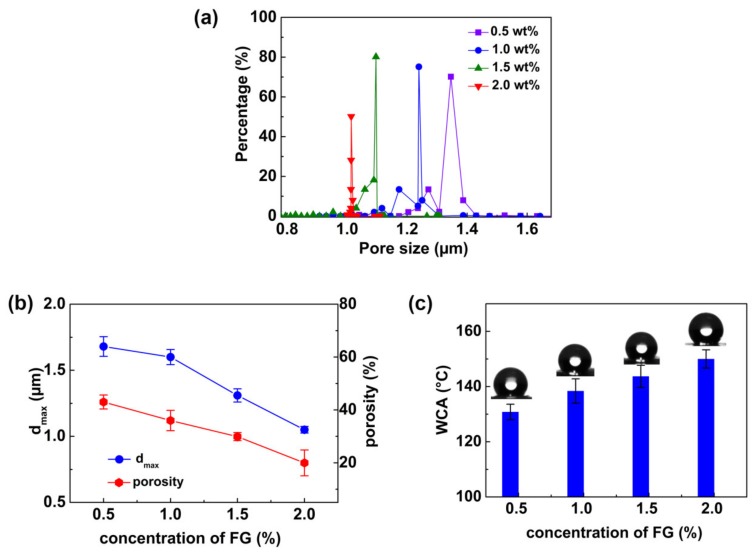
(**a**) Pore size distribution, (**b**) d_max_ and porosity, (**c**) and contact angle of PU/FPU/TPU-50/FG nanofiber WB fabrics at different FG concentrations.

As water droplets will preferentially pass through from larger pores under a certain hydraulic pressure, fibrous membranes with smaller pore diameters would have higher waterproofness [35]. Therefore, the effects of FG on pore size distribution, d_max_ and the porosity of the nanofiber membranes were also investigated (Figure 3a,b). As the FG content increased from 0.5 to 2.0 wt %, the pore size distribution moves to the left, d_max_ of the nanofiber membranes decreased from 1.68 to 1.05 μm, and the porosity decreased from 43% to 20%. These results were caused by the increase of FG content in the nanofiber WB fabrics, which covered the pores of the nanofibers (Figure 2c).

The hydrophobic alkyl chain in FG could affect the surface wettability of the nanofiber WB fabrics. Therefore, the hydrophobicity of the PU/FPU/TPU-50/FG nanofiber WB fabric was investigated by measuring the contact angle. As shown in Figure 3c, as the concentration of FG increased from 0.5 to 2.0 wt %, the water contact angle (WCA) of the nanofiber membrane increased from 130.8° to 150.0°, which indicate that the introduction of FG enabled the PU/FPU/TPU-50/FG surfaces to be more hydrophobic. Such hydrophobic characteristic contributes to the improvement of waterproofness of the nanofiber WB fabrics. 

### 3.3. Waterproofness, Breathability, Antifouling, and Mechanical Performance of PU/FPU/TPU-50/FG WB Fabrics

**Figure 4:**
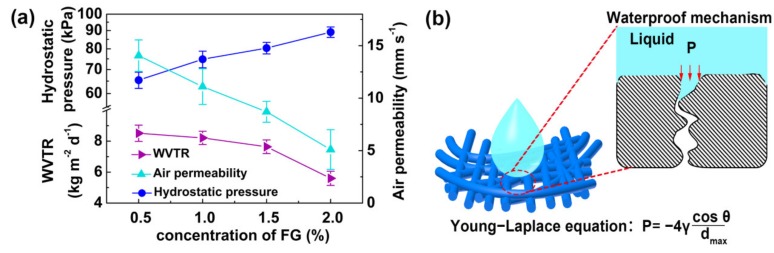
(**a**) Hydrostatic pressure, WVTR, and air permeability of the PU/FPU/TPU-50/FG WB fabrics at different FG concentrations. (**b**) Waterproof mechanism based on the Young−Laplace equation.

The waterproofness and breathability of the nanofiber WB fabrics were evaluated by the hydrostatic pressure, WVTR, and air permeability [36] (Figure 4a). The hydrostatic pressure increased from 65.4 to 89.1 kPa, along with an increase in the FG content from 0.5 to 2.0 wt %. This increasing trend indicates that the waterproofness of the fabric was improved by the introduction of hydrophobic FG (Figure 2c). This was mainly due to the synergistic effect of the decreased d_max_ (Figure 3b) and the increased hydrophobicity (Figure 3c) of the PU/FPU/TPU-50/FG nanofiber surface.

This synergistic effect can be explained by the Young−Laplace equation (Figure 4b), the hydrostatic pressure of the WB fabric was inversely proportional to the d_max_ of the nanofiber membrane and proportional to the contact angle of the hot-pressed membrane. When increasing the content of FG from 0.5 to 2.0 wt %, it was observed that the WVTR decreased from 8.5 to 5.6 kg m^−2^ d^−1^, and the air permeability decreased from 14.1 to 5.1 mm s^−1^, which was due to the reduced porosity.

To meet the demand for practical applications, the PU/FPU/TPU-50/FG-1.5 nanofiber WB fabric was selected as a model to demonstrate waterproofness, breathability and antifouling property. Thus, we carried out a microscopic experiment to demonstrate the waterproofness and breathability (Figure S2), the water droplets in a stand-state were used to demonstrate the waterproofness of the fabric, the silica gel particles were used as a humidity indicator to confirm if water vapor can transferred from the PU/FPU/TPU-50/FG-1.5 nanofiber WB fabric. At a low humidity of 20% and room temperature of 25 °C, the PU/FPU/TPU-50/FG-1.5 nanofiber WB fabric was placed over a beaker, which had been filled with boiling water. Here, the water droplets retained their stand-state on the PU/FPU/TPU-50/FG-1.5 nanofiber WB fabric instead of spreading or evaporating after 30 min standing. Moreover, the color of allochroic silica gel changed from blue to pink (no color was observed until the humidity of the environment increased by 20%), indicating that a large amount of water vapor can be transferred from the fabric within 30 min. The results confirm the good waterproofness and breathability of the PU/FPU/TPU-50/FG-1.5 fabric. Thereafter, water, oil, coffee, and milk were used to contaminate the surface of the obtained fabric (Figure S3). When these droplets dripped onto the fabric, they rolled down smoothly without fouling or wetting the WB fabric surface, indicating the superior antifouling ability of the obtained fabric, and the fabric satisfies the requirements for application in self-cleaning materials, outdoor-sportswear, and chemical-protective clothing [37,38,39].

**Figure 5:**
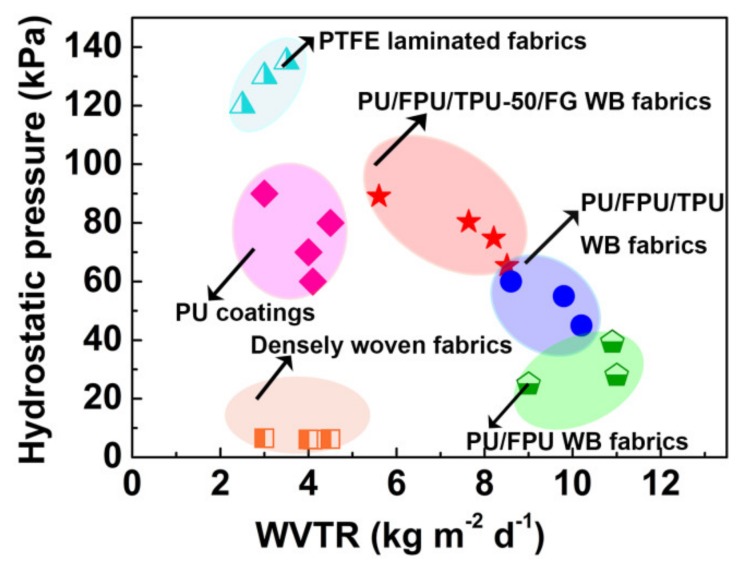
Comparison of waterproofness and breathability of WB fabrics.

**Figure 6:**
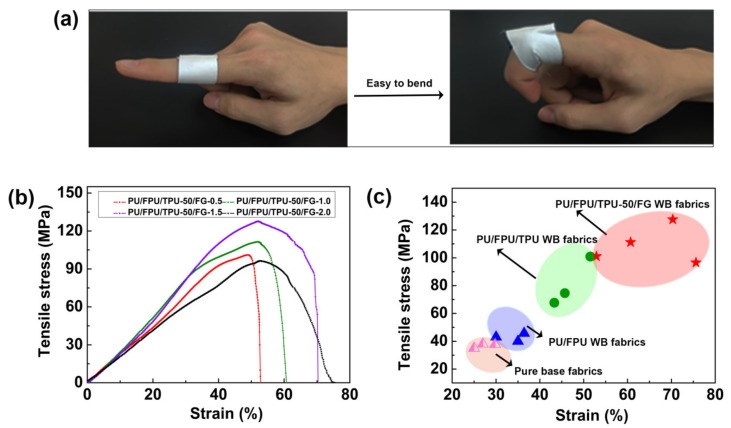
(**a**) Demonstration of the softness of PU/FPU/TPU-50/FG-1.5 WB fabric. (**b**) Stress–strain curve of the PU/FPU/TPU-50/FG WB fabrics at different FG concentrations. (**c**) Mechanical performance of pure base fabrics, PU/FPU WB fabrics, PU/FPU/TPU WB fabrics, PU/FPU/TPU-50/FG WB fabrics.

In general, waterproofness and breathability are two contradictory performance features [40]. Therefore, it is important to maintain a balance between these performances. As illustrated in Figure 5, we compared the waterproofness and breathability among our prepared WB fabrics and other reported records of typical WB fabrics [41,42]. It should be mentioned that in the Figure 5 and Figure 6c, the dots of different shapes represent specific values, and the elliptical area represents the floating range of the values. PTFE laminated fabrics provide the highest hydrostatic pressure (110−140 kPa), but very low WVRT (2.5−3.5 kg m^−2^ d^−1^). PU coatings possess midrange hydrostatic pressure (60−90 kPa), but with a quite low WVTR (<4.5 kg m^−2^ d^−1^). Compared to the two aforementioned fabrics, densely woven fabrics exhibit high WVRT (6.0−6.5 kg m^−2^ d^−1^), but show very low hydrostatic pressure (2.5−3.5 kPa). In dramatic contrast, the prepared PU/FPU, PU/FPU/TPU, PU/FPU/TPU-50/FG WB fabrics display high waterproofness and superior breathability. Among them, PU/FPU/TPU-50/FG WB fabrics has an excellent comprehensiveness of waterproofness and breathability.

As shown in Figure 6a, the prepared nanofiber WB fabric was soft, bendable, and comfortable to wear. In practical applications, strong mechanical performances are required to satisfy the demand for the stretching of the fabric during usage. Therefore, the influence of the FG contents (0.5, 1.0, 1.5, and 2.0 wt %) on the mechanical performance of the PU/FPU/TPU-50/FG nanofiber WB fabrics was investigated. As presented in Figure 6b, when the FG content increased from 0.5 to 1.5 wt %, the tensile stress of the fabric increased from 101.1 to 125.2 MPa, and the strain also increased from 52.9% to 70.3% The improved mechanical performance was ascribed to the adhesion, blending, and overlapping of FG on the surface of the nanofiber (Figure 2a, b and c). More importantly, the existence of FG lowered the smoothness of the nanofiber, which made the fabric difficult to break. When the FG content increased to 2.0 wt %, the tensile stress of the fabric decreased to 96.6 MPa, while the strain increased to 75.6%. This result was obtained because the gradual increase of the FG content in the fabric led to a more fragile characteristic; consequently, the fabric could not stand the large tensile stress. However, the strain of the nanofiber WB fabric increased. This result was attributed to the adhesion, blending, and overlapping of the FG structure in the fabrics [43]. Thereafter, we compared the mechanical performance of pure base fabrics, PU/FPU WB fabrics, and PU/FPU/TPU WB fabrics to PU/FPU/TPU-50/FG WB fabrics. Figure 6c clearly shows that the PU/FPU/TPU-50/FG WB fabrics can provide the tensile stress of more three times and the strain of more two times than the original PU/FPU WB fabric. Moreover, the waterproofness, breathability, and mechanical performance of the fabric could be well maintained after 50 times washing with tap water by drum washing machine (Table S1). These results clearly showed that the PU/FPU/TPU-50/FG nanofiber WB fabrics possessed robust durability [44,45,46]. 

Based on the above results, it could be observed that a PU/FPU/TPU-50/FG nanofiber WB fabric with a high hydrostatic pressure (80.4 kPa), modest WVTR (7.64 kg m^−2^ d^−1^), and robust tensile stress (127.59 MPa) can be obtained when the FG content is 1.5 wt %. Therefore, the addition of hydrophobic FG not only improved the waterproofness but also the mechanical performance of the PU/FPU/TPU-50/FG nanofiber WB fabric.

### 3.4. The Inter-fiber Fusion Structure of PU/FPU/TPU WB Fabrics

**Figure 7:**
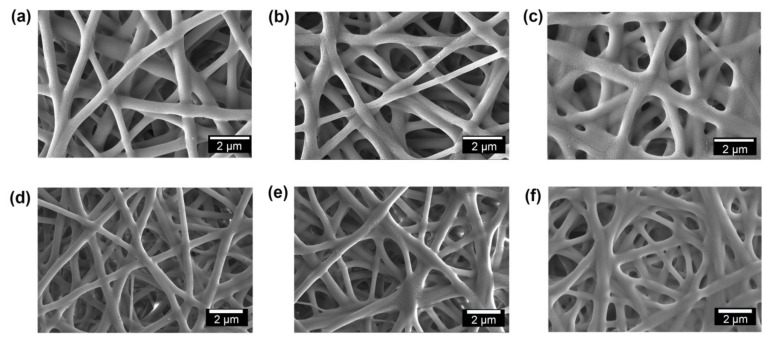
SEM images of the PU/FPU/TPU-50 nanofiber WB fabrics at different heat-treatment temperatures of (**a**) 100, (**b**) 120, and (**c**) 140 °C. SEM images of the PU/FPU/TPU nanofiber WB fabrics at various TPU concentrations: (**d**) 25, (**e**) 50, and (**f**) 75 wt %.

It is expected that proper inter-fiber fusion structure could endow WB fabrics with excellent waterproofness and mechanical performance. Thus, the PU/FPU/TPU-50 WB fabric was selected as a model to investigate the effect of inter-fiber fusion structure on the waterproofness and mechanical performance of WB fabrics. The hot-press temperature and TPU concentration in the PU/FPU/TPU-50 WB fabric are the two important factors that may affect the inter-fiber fusion structure. First, the effect of hot-press temperature on the inter-fiber fusion structure was investigated. At the hot-press temperature of 80 °C, the individual nanofibers could be observed and the pores between adjacent nanofibers were visible (Figure S4). A reasonable explanation for this phenomenon could be that the hot-press temperature did not reach the glass transition temperature of TPU, and therefore the nanofiber did not melt. When the hot-press temperature was 100 °C (slightly higher than the glass transition temperature of TPU), adjacent nanofibers bonded and created an adhesion structure in the nanofiber membrane (Figure 7a), which could be caused by the melting of a small portion of the TPU between the nanofibers. When the hot-press temperature was 120 °C, a significant fiber bonding was observed (Figure 7b), which could be attributed to the fact that sufficient melting occurs between the nanofibers when the hot-press temperature is higher than the glass transition temperature of TPU. Furthermore, when the hot-press temperature was increased to 140 °C, the morphology of the nanofibers suffered serious damage and formed a solid-like membrane (Figure 7c), which could be due to the higher degree of melting of TPU at a high hot-press temperature. 

As shown in Figure S5, when the initial hot-press temperature was 80 °C, the tensile strength of the membrane fabric was 47 MPa, and the breaking elongation was 36.6%. By increasing the temperature from 100 to 120 °C, the tensile strength of the fiber membrane increased from 80.1 to 100.9 MPa, and the breaking elongation also increased from 40.0% to 51.5%. These results could be derived from the increased adhesion degree among the adjacent fibers (Figure 7a,b) at a comparatively high treatment temperature. However, when the hot-press temperature was further increased to 140 °C, the tensile strength and breaking elongation of the nanofiber WB fabric decreased. This result was due to the excessive treatment temperature, which further caused the fusion of the fiber membranes to a solid-like membrane (Figure 7c). The solid-like structure was so fragile that it could not bear the tensile strength [47]. It could be observed that proper hot-press temperature (120 °C, herein) contributes to the improvement of the mechanical performance. 

To ensure the sufficient bonding of the nanofibers, a hot-press temperature of 120 °C was selected for further investigation of the influence of the TPU contents (0, 25, 50, and 75 wt %) on the inter-fiber fusion structure of the PU/FPU/TPU nanofiber WB fabrics. Without TPU in the PU/FPU nanofiber WB fabric, no adhesion structure among the adjacent fibers was observed (Figure S6) because the temperature of the hot-press (120 °C) was not enough to induce the fusion of PU (glass transition temperature: 170−190 °C [48]. When 25 wt % TPU was included in the nanofiber, the surface morphology of the PU/FPU/TPU-25 nanofiber WB fabric exhibited only slight adhesion structures among the adjacent fibers (Figure 7d), which was attributed to the slight fusion of TPU. A further increase of the TPU content to 50 wt % resulted in noticeable adhesion structures among the adjacent nanofibers (Figure 7e), which were caused by the sufficient fusion of TPU. However, when the content of TPU was further increased to 75 wt %, the morphology of the fiber membrane suffered severe damage, and the fiber membrane appeared solid-like (Figure 7f), which could be attributed to the significant fusion of a large amount of TPU [49,50]. It can be observed that, 50% TPU are suitable to induce sufficient inter-fiber fusion structure of the PU/FPU/TPU nanofiber WB at the hot-press temperature of 120 °C.

**Figure 8:**
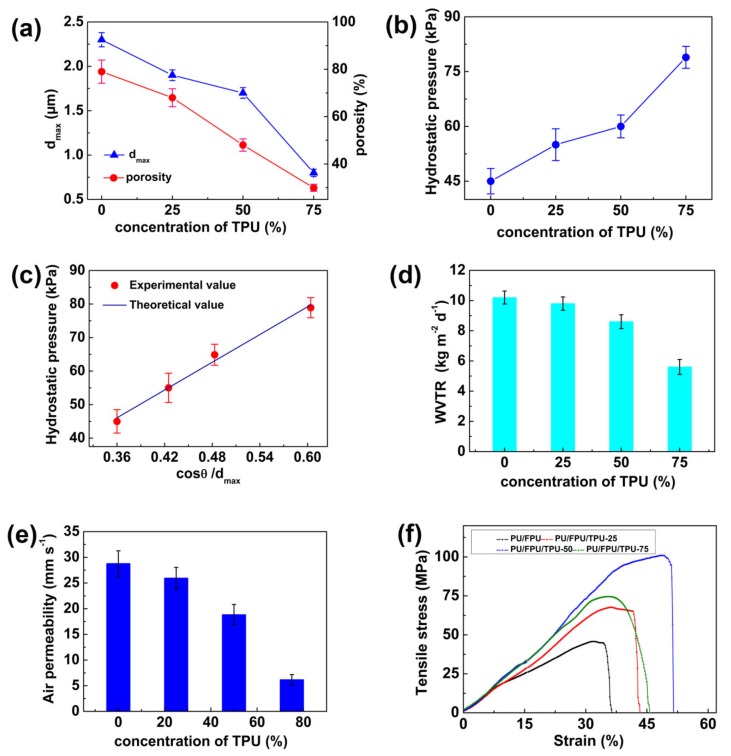
(**a**) dmax and porosity, (**b**) hydrostatic pressure, (**c**) measured and theoretical hydrostatic pressure, (**d**) WVTR, (**e**) air permeability, (**f**) stress–strain curves of PU/FPU/TPU nanofiber WB fabrics at different TPU concentrations.

Furthermore, the effect of the TPU content on the maximum pore size (d_max_) and the porosity of the PU/FPU/TPU series nanofiber WB fabrics was investigated (Figure 8a). The d_max_ of the nanofiber membrane decreased from 2.3 to 0.8 μm with increasing TPU content from 0% to 75%. This result was mainly caused by the increased adhesion structure in the nanofiber membrane. Similarly, the porosity also decreased from 79% to 30% with increasing TPU content from 0% to 75%, which was due to the decrease in the number of pores in the nanofiber membrane with the formation of the adhesion structure among the nanofibers.

According to the Young−Laplace equation, the decrease in d_max_ was in favor of the improvement of waterproofness. The results show that the increment of the TPU content from 0% to 75% resulted in an increase in the hydrostatic pressure from 45.0 to 78.9 kPa (Figure 8b). The measured and theoretical hydrostatic pressure data fit well with the Young−Laplace equation (Figure 8c). By increasing the TPU content from 0% to 75%, the WVTR and air permeability decreased from 10.2 to 5.7 kg m^−2^ d^−1^ and from 28.7 to 6.1 mm s^−1^, respectively, owe to the reduction in porosity (Figure 8d and e).

As shown in Figure 8f, the nanofiber WB fabric demonstrated increased tensile stress from 45.7 to 100.9 MPa and increased stress from 35.8% to 51.5%, with the content of TPU increasing from 0% to 50%. This could because the nanofibers with a relatively high degree of adhesion structures (Figure 7d and e) could bear more load. However, when the concentration of TPU was increased to 75%, the tensile stress and strain of the membrane fabric decreased to 74.7 MPa and 45.7%, respectively. This was mainly due to the very poor flexibility of the adhesion structure (Figure 7f), which could not withstand a large load [51,52,53]. Therefore, the PU/FPU/TPU nanofiber WB with 50% TPU shows the best mechanical performance due to the sufficient inter-fiber fusion structure.

By comparing the inter-fiber fusion structure of PU/FPU/TPU WB fabric, it can be observed that a hot-press temperature of 120 °C and a TPU content of 50 wt % showed a modest hydrostatic pressure (60 kPa), a good WVRT (8.6 kg m^−2^ d^−1^), robust tensile stress (100.9 MPa), and high tensile strain (51.5%), which reflects that the waterproofness and mechanical performance of the PU/FPU/TPU WB fabric with the inter-fiber fusion structure were more than twice than that of the PU/FPU WB fabric without the inter-fiber fusion structure. Therefore, the sufficient fusion structure in the nanofiber WB fabrics did improve the waterproofness and mechanical performance.

## 4. Conclusions

In summary, we have demonstrated a simple and feasible strategy for preparing high-performance WB fabrics with scalability, by combining electrospinning and hot-press treatment technology. This work systematically optimized the waterproofness, breathability and mechanical performance of the PU/FPU/TPU-50/FG WB fabrics by adjusting the concentrations of FG and TPU as well as the hot-press temperature. More importantly, the introduction of the hydrophobic FG sheet structure and the formation of the inter-fiber fusion structure by TPU greatly improved not only the waterproofness but also the mechanical performance of the nanofiber WB fabrics. The obtained PU/FPU/TPU-50/FG-1.5 nanofiber WB fabric exhibited an excellent hydrostatic pressure (80.4 kPa), which significantly exceeded the prepared PU/FPU, PU/FPU/TPU WB fabrics, and most WB fabrics recorded in the aforementioned report, and a modest WVTR (7.64 kg m^−2^ d^−1^), which was greater than that of the typical WB fabrics. Moreover, compared with the original PU/FPU WB fabrics and pure base fabric, the PU/FPU/TPU-50/FG-1.5 nanofiber WB fabric demonstrated robust tensile stress (127.59 MPa) and strain of 70.3%, making it a very promising candidate for high-performance WB materials due to its excellent comprehensive performance and easy mass production. The PU/FPU/TPU-50/FG-1.5 nanofiber WB fabric has potential applications in self-cleaning materials, outdoor-sportswear, and chemical-protective clothing.

## Supplementary Materials

Supplementary Materials can be found at https://www.mdpi.com/2073-4360/12/4/836/s1. Figure S1: Diameter distribution diagram of PU/FPU/TPU-50/FG-0.5 nanofiber WB fabric, Figure S2: Demonstration waterproofness and breathability of PU/FPU/TPU-50/FG-1.5 WB fabric, Figure S3: Lyophobic property of PU/FPU/TPU-50/FG-1.5 WB fabric. Table S1: Washing fastness of the PU/FPU/TPU-50/FG-1.5 fabric, the data was obtained after 50 times washing. Figure S4: (a) SEM image of the PU/FPU/TPU-50 membranes at a hot-press temperature of 80 °C. Figure S5: Tensile strength and elongation of the PU/FPU/TPU-50 nanofiber WB fabric at different heating temperatures. Figure S6: SEM image of the PU/FPU membranes at a hot-press temperature of 120 °C.
