# Supplementary material for: Hot-melt Adhesive Bonding of Polyurethane/Fluorinated Polyurethane/Alkylsilane-Functionalized Graphene Nanofibrous Fabrics with Enhanced Waterproofness, Breathability, and Mechanical Properties
Source: Polymers (Basel). 2020 Apr 6;12(4):836. doi: 10.3390/polym12040836 (PMC7240538; doi:10.3390/polym12040836)
Supplement: Supplementary file 1 [file polymers-12-00836-s001.pdf]

Supplementary Material

Hot-melt adhesive bonding of polyurethane/fluorinated polyurethane/alkylsilane-functionalized graphene nanofibrous fabrics with enhanced waterproofness, breathability, and mechanical properties

Chunhui Liu[1], Xi Liao[1], Weili Shao[1], Fan Liu[1], Bin Ding[2], Gaihuan Ren[1]*, Yanyan Chu[1]*, and Jianxin He[1]**

[1] College of Textile, Zhongyuan University of Technology, Henan province, Zhengzhou 450007, China; lch1820921715@163.com (C.L.); xiliao1208@163.com (X.L.); WeiliShao@163.com (W.S.); liufan_a@hotmail.com (F.L.)

[2] Key Laboratory of Textile Science & Technology, Ministry of Education, College of Textiles,Donghua University, Shanghai 201620, China; binding@dhu.edu.cn

* Correspondence: hejianxin771117@163.com (J.H.); 6018@zzti.edu.cn (Y.C.); ghren@zut.edu.cn (G.R.)

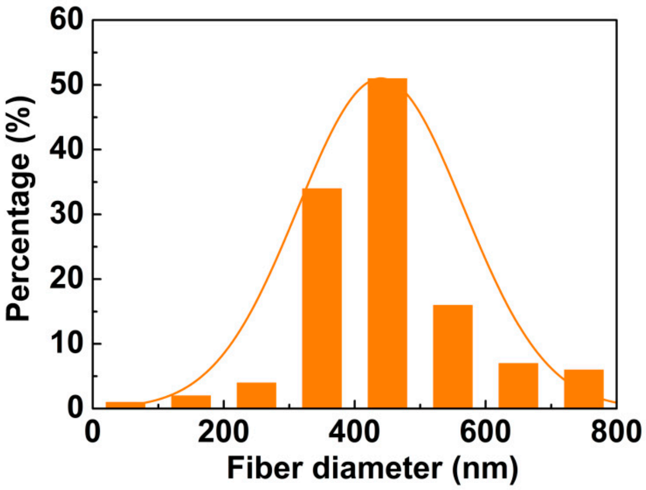

Figure S1. Diameter distribution diagram of PU/FPU/TPU-50/FG-0.5. nanofiber WB fabric

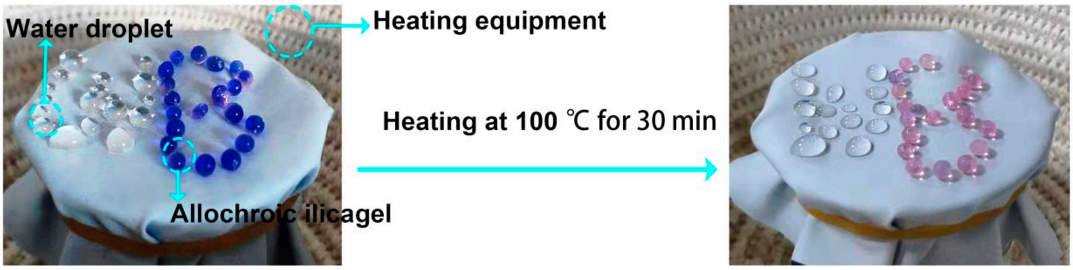

Figure S2. Demonstration waterproofness and breathability of PU/FPU/TPU-50/FG-1.5 WB fabric.

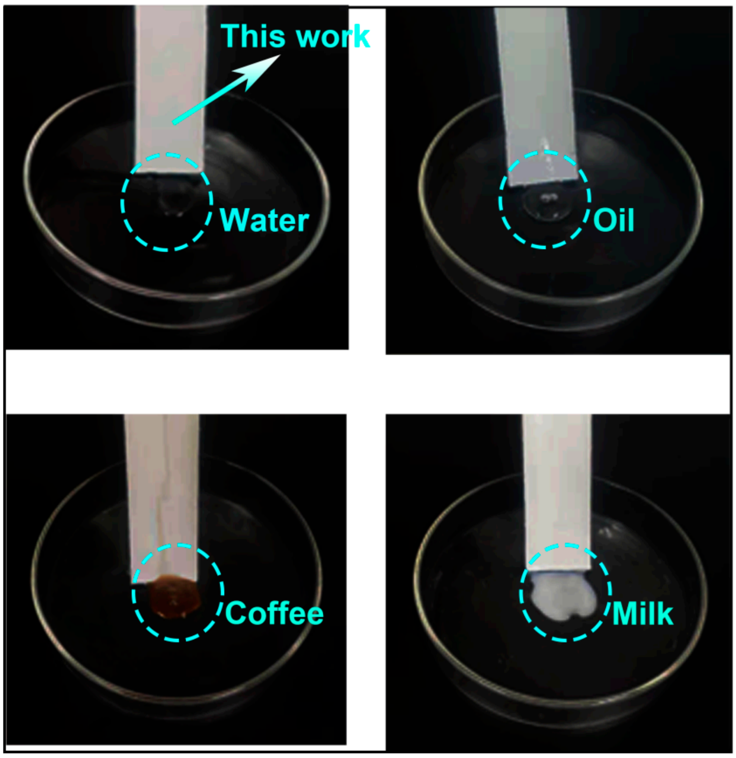

Figure S3. Lyophobic property of PU/FPU/TPU-50/FG-1.5 WB fabric.

Table S1. Washing fastness of the PU/FPU/TPU-50/FG-1.5 fabric, the data was obtained after 50 times washing by drum washing machine.

Samples	Hydrostatic pressure (kPa)	WVRT (kg m^{-2} d^{-1})	Tensile stress (MPa)
PU/FPU/TPU-50/FG-1.5	78.6±3.0	8.3±0.5	119.64±2.5

Samples	WCA (°)	Air permeability (mm s^{-1})	Strain (%)
PU/FPU/TPU-50/FG-1.5	140.1±2.5	10.6±1.5	68.5±2.0

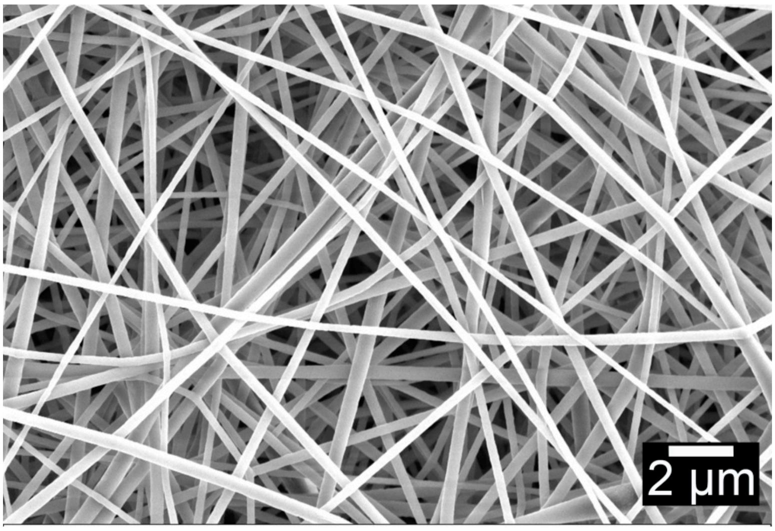

Figure S4. (a) SEM image of the PU/FPU/TPU-50 membranes at a hot-press temperature of 80 °C.

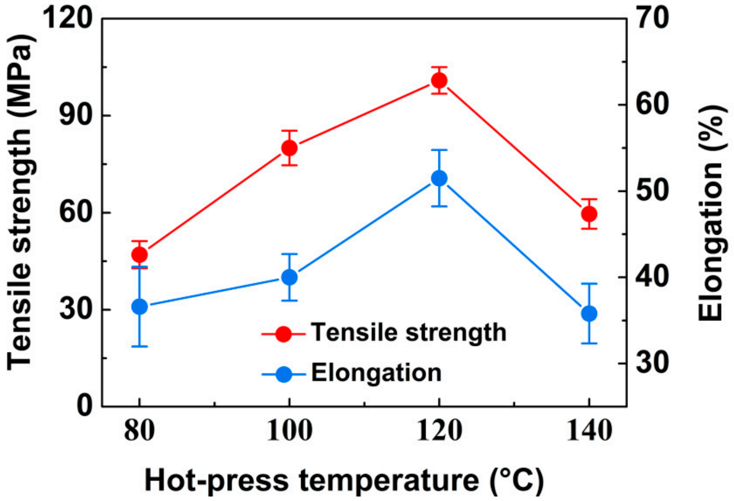

Figure S5. Tensile strength and elongation of the PU/FPU/TPU-50 nanofiber WB fabric at different heating temperatures.

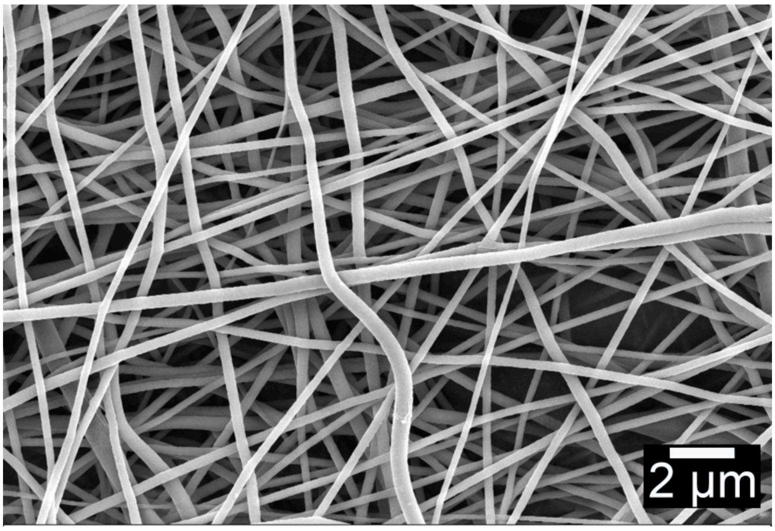

Figure S6. SEM image of the PU/FPU membranes at a hot-press temperature of 120 °C.
